# Molecular Epidemiology of Multidrug-Resistant *Klebsiella pneumoniae* Isolates in a Brazilian Tertiary Hospital

**DOI:** 10.3389/fmicb.2019.01669

**Published:** 2019-07-23

**Authors:** Jussara Kasuko Palmeiro, Robson Francisco de Souza, Marcos André Schörner, Hemanoel Passarelli-Araujo, Ana Laura Grazziotin, Newton Medeiros Vidal, Thiago Motta Venancio, Libera Maria Dalla-Costa

**Affiliations:** ^1^Laboratório de Bacteriologia e Biologia Molecular, Unidade do Laboratório de Análises Clínicas, Complexo Hospital de Clínicas, Universidade Federal do Paraná, Curitiba, Brazil; ^2^Faculdades Pequeno Príncipe, Instituto de Pesquisa Pelé Pequeno Príncipe, Curitiba, Brazil; ^3^Departamento de Análises Clínicas, Centro de Ciências da Saúde, Universidade Federal de Santa Catarina, Florianópolis, Brazil; ^4^Laboratório de Estrutura e Evolução de Proteínas, Instituto de Ciências Biomédicas II, Universidade de São Paulo, São Paulo, Brazil; ^5^Hospital Universitário, Universidade Federal de Santa Catarina, Florianópolis, Brazil; ^6^Laboratório de Química e Função de Proteínas e Peptídeos, Centro de Biociências e Biotecnologia, Universidade Estadual do Norte Fluminense Darcy Ribeiro, Campos dos Goytacazes, Brazil; ^7^Departamento de Bioquímica e Imunologia, Instituto de Ciências Biológicas, Universidade Federal de Minas Gerais, Belo Horizonte, Brazil; ^8^National Center for Biotechnology Information, National Library of Medicine, National Institutes of Health, Bethesda, MD, United States

**Keywords:** Brazil, hospital outbreak, MLST, antimicrobial resistance, clonal group 258, whole-genome sequencing

## Abstract

Multidrug-resistant (MDR) *Klebsiella pneumoniae* (Kp) is a major bacterial pathogen responsible for hospital outbreaks worldwide, mainly via the spread of high-risk clones and epidemic resistance plasmids. In this study, we evaluated the molecular epidemiology and β-lactam resistance mechanisms of MDR-Kp strains isolated in a Brazilian academic care hospital. We used whole-genome sequencing to study drug resistance mechanisms and their relationships with a *K. pneumoniae* carbapenemase-producing (KPC) Kp outbreak. Forty-three Kp strains were collected between 2003 and 2012. Antimicrobial susceptibility testing was performed for 15 antimicrobial agents, and polymerase chain reaction (PCR) was used to detect 32 resistance genes. Mutations in *ompk35*, *ompk36*, and *ompk37* were evaluated by PCR and DNA sequencing. Pulsed field gel electrophoresis (PFGE) and multilocus sequence typing (MLST) were carried out to differentiate the strains. Based on distinct epidemiological periods, six Kp strains were subjected to whole-genome sequencing. β-lactamase coding genes were widely distributed among isolates. Almost all isolates had mutations in porin genes, particularly *ompk35*. The presence of *bla*_KPC_ promoted a very high increase in carbapenem minimum inhibitory concentration only when *ompk35* and *ompk36* were interrupted by insertion sequences. A major cluster was identified by PFGE analysis and all isolates from this cluster belonged to clonal group (CG) 258. We have also identified a large repertoire of resistance genes in the sequenced isolates. A *bla*_KPC–2_-bearing plasmid (pUFPRA2) was also identified, which was very similar to a plasmid previously described in the first Brazilian KPC-Kp (2005). We found high-risk clones (CG258) and an epidemic resistance plasmid throughout the duration of the study (2003 to 2012), emphasizing a persistent presence of MDR-Kp strains in the hospital setting. Finally, we found that horizontal transfer of resistance genes between clones may have played a key role in the evolution of the outbreak.

## Introduction

Multidrug-resistant *Klebsiella pneumoniae* (MDR-Kp) is recognized in healthcare settings as a cause of high morbidity and mortality among patients with severe infections. Some MDR-Kp isolates have evolved to become extensively drug-resistant (XDR) isolates that have few therapeutic options (Lee et al., 2016). In Brazil, the National Program for Monitoring Bacterial Resistance has reported increasing annual rates of carbapenem-resistant Kp isolated from bloodstream infections (Anvisa, 2017). Carbapenem resistance is attributed to a high expression of carbapenemases and extended spectrum β-lactamases (ESBLs) or AmpC β-lactamases coupled with modification of outer membrane permeability (Fernandez and Hancock, 2012). Kp produces an intrinsic β-lactamase, *bla*_SHV_, and two major porins, OmpK35 and OmpK36, in addition to the major multidrug efflux pump AcrAB-TolC, which may also be related to this phenotype (Fernandez and Hancock, 2012; Lee et al., 2016).

*Klebsiella pneumoniae* carbapenemase-producing Kp (KPC-Kp) is a major bacterial pathogen responsible for hospital outbreaks worldwide (Lee et al., 2016), mainly via the spread of high-risk clones and epidemic resistance plasmids (Mathers et al., 2015). In general, these clones belong to clonal group 258 (CG258), which comprises 43 different sequence types (STs) (Chen et al., 2014) between single- and double-locus variants, based on multilocus sequence typing (MLST) (Chen et al., 2014; Bowers et al., 2015; Gaiarsa et al., 2015). Epidemiological data have reported that STs 11, 258, 340, 437, and 512 comprise most of the *bla*_KPC_ CG258 isolates (Chen et al., 2014; Bowers et al., 2015; Gaiarsa et al., 2015). Furthermore, epidemic resistance plasmids harboring *bla*_CTX–M_ and *bla*_KPC_, often belong to incompatibility groups F and N and are common among members of the STs from CG258 (Mathers et al., 2015; Lee et al., 2016).

Here, we evaluated the molecular epidemiology and β-lactam resistance mechanisms of MDR-Kp strains isolated between 2003 and 2012 in a Brazilian academic care hospital. We also selected six MDR-Kp strains for whole-genome sequencing (WGS) to gather insights on their drug resistance mechanisms and association with a KPC-Kp outbreak.

## Materials and Methods

### Study Setting

This study was performed at Complexo Hospital de Clínicas of the Universidade Federal do Paraná (CHC/UFPR), a 655-bed tertiary hospital located in Curitiba, Paraná, Southern Brazil. CHC/UFPR is a referral center which also supports other hospitals. The Institutional Ethics Review Board of the CHC/UFPR approved this study under reference number IRB#: 2656.263/2011-11.

### Bacterial Strains and Phenotypic Tests

A total of 43 clinical isolates of Kp from different body sites of 32 patients were studied. These isolates were selected from a CHC-UFPR bacterial collection. Only isolates resistant to at least one carbapenem (ertapenem) by disk diffusion testing were included. These isolates were collected between August 2003 and February 2012, a time interval that we divided into three well-defined epidemiological periods, according to the prevalence of MDR-*Enterobacteriaceae*. The first period (2000–2009) was characterized by ESBL prevalence, resistance to fluoroquinolones and aminoglycosides, and low resistance to imipenem and meropenem (Nogueira Kda et al., 2014, Nogueira et al., 2015). The second period was defined by a KPC-Kp outbreak that occurred in June 2010 (Almeida et al., 2014), and the third period was characterized by a gradual increase in the prevalence of KPC-Kp and other *Enterobacteriaceae*.

**FIGURE 1:**
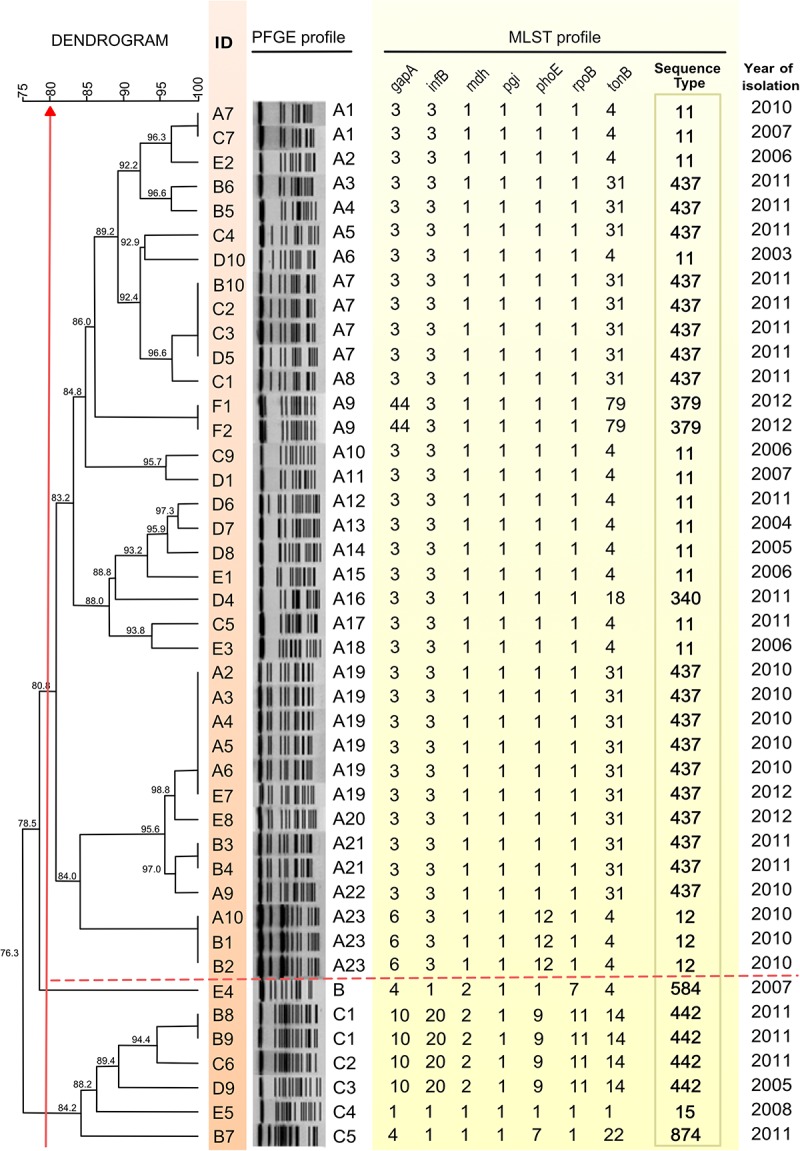
Dendrogram constructed on the basis of PFGE patterns and MLST profile of 43 *K. pneumoniae* isolates. A dice coefficient similarity of at least 80% included two PFGE clusters designated as A and C, as indicated by the vertical red arrow crossing the dendrogram on the left. Isolate identifiers are shown aligned to the dendrogram tips in the column ID. A dashed line delimits the cluster A, which contains the largest numbers of PFGE profiles. Isolates with the same pulsotype designation (column PFGE profile) are genetically indistinguishable under this procedure. KpA2, KpA3, KpA4, KpA5, KpA6, and KpA9 are isolates of the Kp outbreak.

Five isolates recovered from patients treated in four other hospitals were also included (C4, C5, C7, D1, and D5; Figure 1 and Table 1). In all but six cases, a single bacterial specimen was isolated. However, from each of those six patients, between two and four bacterial samples were isolated (Table 1). Kp isolates recovered from clinical specimens were stored at −80°C in trypticase soy broth (TSB; HiMedia, Mumbai, India) containing 15% glycerol. Bacterial isolates were identified using a Vitek2 Compact instrument (BioMérieux S.A., Marcy l’Etoile, France) and mass spectrometer (Microflex LT; Bruker Daltonics, Bremen, Germany).

**TABLE 1:** Clinical data, antibiotic susceptibilities and molecular features of 43 *K. pneumoniae* isolates^a–d^.

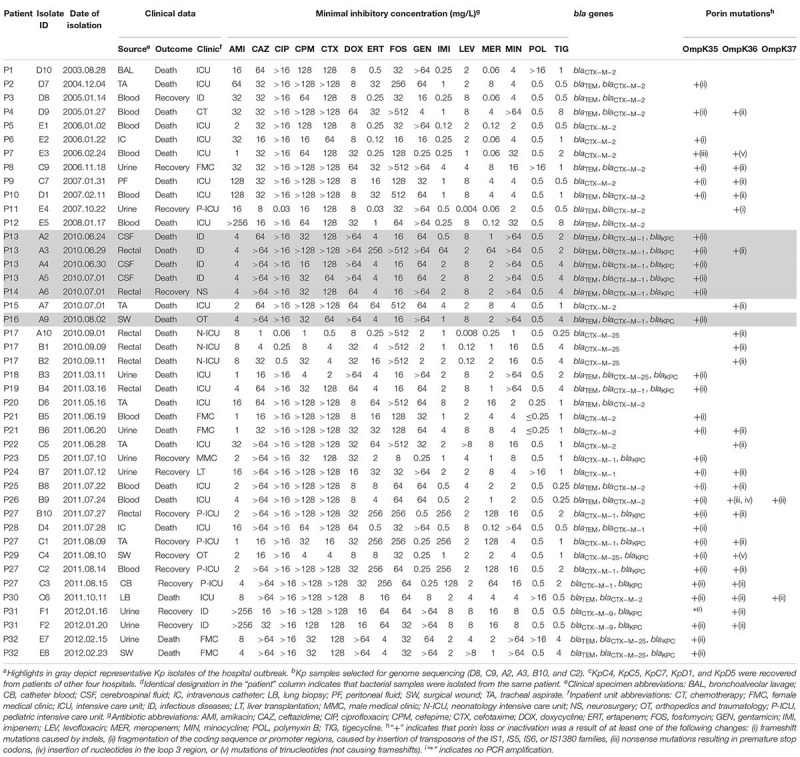

Antimicrobial susceptibility testing (AST) was performed for 15 antimicrobial agents (Table 1) by agar dilution, except for polymyxin which was tested by broth dilution, as recommended by the Clinical and Laboratory Standards Institute (CLSI). Minimal inhibitory concentrations (MICs) were interpreted according to CLSI standards (CLSI M100-S27 document, 2017^1^). Polymyxin, tigecycline, and fosfomycin breakpoints were interpreted using Brazilian Committee on AST and European Committee on AST standards (BrCAST-EUCAST^2^). Double-disk synergy (EUCAST, 2013^3^) and imipenem hydrolysis assay by spectrophotometry (Nicoletti et al., 2015) were performed to determine the carbapenem resistance phenotypes.

### Antibiotic Resistance Characterization and Molecular Typing

The presence of *bla*_*MOX*_, *bla*_CMY_, *bla*_LAT_, *bla*_BIL_, *bla*_DHA_, *bla*_ACC_, *bla*_*MIR*_, *bla*_ACT_, *bla*_*FOX*_, *bla*_TEM_, *bla*_CTX–M–1,__–M–2,__–*M*–8,__–M–9,__–M–25_, *bla*_PER_, *bla*_BES_, *bla*_VEB_, *bla*_KPC_, *bla*_*GES*_, *bla*_IMP_, *bla*_VIM_, *bla*_NDM_, *bla*_SPM_, *bla*_*GIM*_, *bla*_SIM,_ *bla*_OXA–23_, *bla*_OXA–48_, *bla*_OXA–51_, *bla*_OXA–58_, *bla*_OXA–143_, and *bla*_BKC_ was investigated by polymerase chain reaction (PCR) using primers and amplification conditions indicated in Supplementary Table 1.

Mutations in *ompk35*, *ompk36*, and *ompk37* were evaluated by PCR and DNA sequencing (Kaczmarek et al., 2006; Nicoletti et al., 2015). PCR products were sequenced using a 3730XL DNA Analyzer (Applied Biosystems, Carlsbad, CA, United States). Nucleotide and protein sequences were compared to the reference proteins OmpK35 (GenBank accession no. AJ011501), OmpK36 (accession no. Z33506), and OmpK37 (accession no. AJ011502). Genes or promoter regions of porins truncated by insertion sequences (IS) were evaluated using ISFinder (Siguier et al., 2006).

Pulsed-field gel electrophoresis (PFGE) was performed according to Nogueira et al. (Nogueira Kda et al., 2014; Nogueira et al., 2015) to differentiate between isolates. Gels were analyzed with BioNumerics program version 6.6 (Applied Maths, Kortrijk, Belgium). The dice similarity coefficient was used to determine the similarity between each banding pattern. A dendrogram was constructed using the unweighted-pair group method with arithmetic averages. The values used for optimization and tolerance were 1.0 and 2.0%, respectively. Isolates with similarities greater than 80% were considered to belong to the same cluster, following previously proposed criteria (Tenover et al., 1995). Different PFGE profiles within clusters were numbered according to the order in the dendrogram (Figure 1). MLST was performed by PCR and sequencing of seven Kp housekeeping genes (i.e., *gapA*, *infB*, *mdh*, *pgi*, *phoE*, *rpoB*, and *tonB*) following the protocol available at the Pasteur MLST website (Diancourt et al., 2005).

### Genome Sequencing, Assembly, and Annotation

Based on three previously defined epidemiological periods, antimicrobial resistance profiles, and body sites from which strains were isolated, six Kp isolates were selected for genome sequencing, including the index isolates KpA2 and KpA3 of the KPC outbreak. KpD8/KpC9 and KpB10/KpC2 were isolated before and after the outbreak, respectively (Table 1). KpA2 and KpA3 were isolated from different body sites of the same patient (P13), as were KpB10 and KpC2 (P27), while C9 and D8 were obtained from distinct patients (P8 and P3, respectively) (Table 1). Genomic DNA was extracted using a DNeasy 96 Blood & Tissue Kit (Qiagen, Silicon Valley, CA, United States) and sequenced at the Life Sciences Core Facility of the State University of Campinas (LaCTAD; São Paulo, Brazil).

Paired-end (PE) libraries with an average insert size of 550 bp fragments were generated using an Illumina TruSeq DNA PCR-free LT Kit (Illumina Inc., San Diego, CA, United States) and sequenced (PE, 2 × 150 bp) using a HiSeq 2500 instrument in RAPID run mode (Illumina Inc.).

Quality-based trimming and filtering were performed using Trimmomatic version 0.32 (Bolger et al., 2014). Paired-end reads were assembled using Velvet version 1.2.10 (Zerbino and Birney, 2008). Chromosomal and plasmid contigs were manually inspected and separated based on BLASTn results, considering the best hit for identity and coverage. Chromosomal contigs were scaffolded using SSPACE version 3.0 (Boetzer et al., 2011). To sort the chromosomal sequence, the scaffolds were ordered by synteny against a reference chromosome using Gepard version 5.0 (Krumsiek et al., 2007). For each isolate, the reference genome used for scaffold sorting was the publicly available genome with the most similar *k-*mer spectrum, which was determined by KmerFinder version 2.0^4^, which was Kp HS11286 (GenBank accession no. CP003200.1) (Bi et al., 2015) for KpA2, KpA3, and KpD8 and Kp JM45 (accession no. CP006656.1) for KpB10, KpC2, and KpC9. Gaps within scaffolds were filled using GapFiller version 2.1.1 (Nadalin et al., 2012) and inspected by aligning PE reads against the scaffolds using Bowtie2 version 2.1.0 (Langmead and Salzberg, 2012). Draft chromosomes and plasmid contigs had their genes predicted with Prokka version 1.12 (Seemann, 2014). *In silico* sequence typing was defined by MLST version 1.8^5^.

The presence of plasmids was also investigated using plasmidSPAdes version 3.10.0 (Antipov et al., 2016). The plasmid scaffolds obtained with plasmidSPAdes were compared against all plasmids available in GenBank (Updated 2016.11.03) and plasmid *rep* genes available in PlasmidFinder version 1.3^6^. We also used Bandage (Wick et al., 2015) to analyze graph structures (Supplementary Table 2). Furthermore, plasmids recognized by plasmidSPAdes were mapped against reads and contigs using GFinisher (Guizelini et al., 2016) to improve plasmid assemblies. The complete plasmid was annotated with Prokka and manually curated using similarity with sequences available in UniRef90^7^. Plasmid incompatibility groups were predicted using PlasmidFinder (Supplementary Table 2) and *oriT* region was annotated using oriTfinder (Li et al., 2018).

### Profiling of Antibiotic Resistance-Related Genes

Chromosomal and plasmid antibiotic resistance genes were predicted by ResFinder database version 2.1^8^ and Comprehensive Antimicrobial Resistance Database (CARD) version 1.1.8 (Jia et al., 2017). The Short Read Sequence Typing (SRST2) version 0.2.0 (Inouye et al., 2014) and Genefinder algorithms (Sadouki et al., 2017) were tested to detect resistance genes with both databases. Furthermore, for ResFinder, the following parameters were defined: all databases were set for the antimicrobial configuration, and the type of input was set to assembled genomes/contigs and minimum thresholds of 98% identity and 80% alignment coverage between query and hit sequences.

### Nucleotide Sequence Accession Numbers

The genomes of the six MDR-*K. pneumoniae* subsp. *pneumoniae* isolates have been deposited at DDBJ/ENA/GenBank under the accession numbers: PYWQ00000000 (D8), PYWR00000000 (C9), PYWS00000000 (C2), PYWT00000000 (B10), PYWU00000000 (A3), and PYWV00000000 (A2). The complete nucleotide sequence of the pUFPRA2 plasmid was included under accession number PYWV00000000.

## Results

### Clinical Patient Profiles

Patient outcomes and clinical data are summarized in Table 1. Blood (*n* = 10/43, 23%), urine (*n* = 9/43, 20%), rectal (*n* = 7/43, 16%), and tracheal aspirate (*n* = 5/43, 11%) specimens yielded the highest numbers of isolates. Most patients were in the intensive care unit (ICU), and a high mortality rate was observed (24 out of 32 patients died; Table 1).

### Antimicrobial Susceptibility, β-Lactam Resistance Profile, and Molecular Typing

Table 1 summarizes the results of ASTs. All isolates (except A10 and E4) displayed increased MICs for at least three classes of antibiotics and were classified as MDR (Magiorakos et al., 2012). Nine isolates exhibited sensitivity to all carbapenems by agar dilution.

All Kp isolates had *bla*_CTX–M_ and co-occurrence of *bla*_TEM_ and *bla*_CTX–M_ was found in 48.8% (*n* = 21/43) of isolates. No class C β-lactamase or minor-ESBL (BES, GES, PER, and VEB) was detected. Among carbapenemases, 18 isolates possessed *bla*_KPC_, although no class B or D carbapenemases were detected. Ciprofloxacin and gentamicin showed low activity against ESBL-producing isolates. For isolates co-producing ESBL and KPC, neither ciprofloxacin nor minocycline were effective. All isolates were resistant to doxycycline. Only amikacin, fosfomycin, polymyxin, and tigecycline showed good activity against KPC-ESBL-coproducing isolates.

Nearly 90% of isolates had mutations in porins (*n* = 38/43, Table 1); among them, 33 were carbapenem-resistant and five were carbapenem-sensitive (i.e., A10, D4, E2, E3, and E4). Out of the five remaining samples which did not show porin mutations, four were carbapenem-sensitive (D8, D10, E1, and E5) and one was carbapenem-resistant (D6). Mutations in either *ompk35* or *ompk36* were observed in 14 strains and 6 strains, respectively, while 18 isolates were identified as having mutations in both of these porin genes. Only two isolates had mutations in *ompk35*, *ompk36*, and *ompk37* (Table 1). Types of mutations identified in the porin genes included: frameshift mutations caused by indels (9 isolates), fragmentation of the coding sequence or promoter regions caused by insertion of the IS1-like, IS5-like, IS6-like, or IS1380-like transposons (34 isolates), nonsense mutations resulting in premature stop codons (2 isolates), insertion of nucleotides in the loop 3 region (1 isolate), and mutations of trinucleotides not causing frameshifts (2 isolates) (Table 1 and Supplementary Figure 1).

The o*mpk37* truncation by an IS5-like IS did not result in increased carbapenem MICs (i.e., B9 and C6, Table 1). Moreover, higher carbapenem MICs were observed only when *bla*_KPC_ was associated to *ompk35* and *ompk36* interrupted by ISs. Different antimicrobial resistance profiles were observed in Kp isolated from different body sites of the same patient (Table 1; P13, P17, P21, P27, P31, and P32), justifying their inclusion in the study. In some of these patients, isolates from different body sites had the same *bla* genes, but a different set of porin mutations.

Two distinct clusters A and C (>80% similarity) were identified based on similarities observed in dendrogram analysis based on PFGE typing (Figure 1). Notably, the major part of cluster A isolates (*n* = 33) belong to CG258 (ST11, *n* = 12; ST340, *n* = 1; ST379, *n* = 2; and ST437, *n* = 18), except for three non-CG258 isolates (ST12, *n* = 3). The cluster C displayed STs that do not belong to CG258 (ST15, 442, 584, and 874) (Figure 1). KpB3, KpB4, KpE7, and KpE8 isolates showed more than 95% similarity to outbreak isolates, although these strains were isolated in 2011 and 2012.

### Genomic Diversity of Six Kp Isolates

Pulsed field gel electrophoresis results were not used to select samples for WGS, since most of them belong to a single cluster (cluster A). We performed WGS of six Kp strains from the previously defined epidemiological period and diversity of antimicrobial resistance: two strains isolated before the outbreak, with low (KpD8) and high (KpC9) carbapenem MIC; two strains from the outbreak, with low (KpA2) and high (KpC9) carbapenem MIC (KpA3), and two strains isolated after the outbreak, both with high carbapenem MIC (KpB10 and KpC2) (Table 1).

Each of the six sequenced isolates belonged to cluster A (Figure 1) and had the following distinct PFGE and MLST profiles: KpD8 (pulsotype A14, ST11), KpC9 (pulsotype A10, ST11), KpA2 and KpA3 (pulsotype A19, ST437), and KpB10 and KpC2 (pulsotype A7, ST437). A summary of the genomic features of the six MDR-Kp isolates is shown in Supplementary Table 3.

Resistance genes were widely distributed among isolates. In Table 2, we list resistance genes in the plasmids and chromosomes of the sequenced genomes, which were identified based on the results of manually inspected BLAST searches (see section “Materials and Methods” for details). In addition to the β-lactamases detected by PCR, narrow-spectrum oxacillinases were also found (*bla*_OXA–1_ and *bla*_OXA–2_). No discrepancies were found between PCR and genome sequencing data. Mutations in *ompk35* and *ompk36* were confirmed and mutations in *ompk26*, *lamB*, and *phoE* were not found. Various aminoglycoside-modifying enzymes (AMEs) were detected, even in isolates that showed sensitivity to amikacin and gentamicin (Table 2). However, this result was not supported by all used prediction tools, as we found some divergences in the identification of AMESs from ResFinder, CARD, SRST2, and Genefinder. Determinants of resistance to fluoroquinolones were: (i) mutations in *gyrA* and *parC*, (ii) presence of the acetyltransferase, AAC(6′)Ib-cr, and (iii) presence of *qnrB1* (Table 2). Resistance to levofloxacin emerged when there were more mutations in *gyrA* (Ser83Ile and Asp87Gly; in D8) or when QnrB was present (A2). KpC9 was unique regarding its resistance to polymyxin because the *mgrB* from this isolate was truncated by IS*Kpn13* (IS5 family), which was inserted in the opposite orientation, between nucleotides 75 and 76.

**TABLE 2:** Resistance gene repertoire identified using ResFinder and CARD databases.

**Sample ID**	**KpD8**	**KpC9**	**KpA2**	**KpA3**	**KpB10**	**KpC2**
**Plasmid-mediated**						
Beta-lactams	*bla*_TEM–1_	*bla*_TEM–1_	*bla*_TEM–1_	*bla*_TEM–1_	*bla*_OXA–1_	*bla*_OXA–1_
	*bla*_CTX–M–2_	*bla*_OXA–2_	*bla*_OXA–1_	*bla*_OXA–1_	*bla*_CTX–M15_	*bla*_CTX–M15_
		*bla*_CTX–M–2_	*bla*_CTX–M–15_	*bla*_CTX–M–15_	*bla*_KPC–2_	*bla*_KPC–2_
			*bla*_KPC–2_	*bla*_KPC–2_		
Aminoglycosides	*aac*(*3*)*-IIa*	*aac*(*3*)*-IIa*	*aac*(*3*)*-IId*	*aac*(*3*)*-IId*	*aadA2*	*aadA2*
	*aadA1*(2 copies)	*aadA2*	*aph*(*3*′)*-Ia*	*aph*(*3*′)*-Ia*	*aph*(*3*′)*-Ia*	*aph*(*3*′)*-Ia*
	*aadA2*					
	*aph*(*3*′)*-Ia*					
	*aph*(*3*′)*-VIa*					
Quinolones		*aac*(*6*′)*Ib-cr*	*aac*(*6*′)*Ib-cr*	*aac*(*6*′)*Ib-cr*	*aac*(*6*′)*Ib-cr*	*aac*(*6*′)*Ib-cr*
			*qnrB1*			
Fosfomycin	*fosA5/6*	*fosA5/6*	*fosA5/6*	*fosA5/6*	*fosA5/6*	*fosA5/6*
Sulphonamide	*sul1*	*sul1*	*sul1*	*sul1*	*sul1*	*sul1*
	*sul3*					
Trimethoprim	*dfrA12*	*dfrA12*	*dfrA5*	*dfrA5*	*dfrA5*	*dfrA5*
			*dfrA30*	*dfrA30*	*dfrA8*	*dfrA8*
					*dfrA30*	*dfrA30*
Chloramphenicol	*catA1*		*catB3*	*catB3*	*catA1*	*catA1*
	*cmlA1*				*catB3*	*catB3*
**Chromosome-mediated**						
Beta-lactams	*bla*_SHV–11_	*bla*_SHV–11_	*bla*_SHV–11_	*bla*_SHV–11_	*bla*_SHV–11_	*bla*_SHV–11_
				*bla*_CTX–M–15_		
		*ompk35*, frameshift (Δ342C)	*ompk35*, disrupted by IS	*ompk35*, disrupted by IS	*ompk35*, disrupted by IS	*ompk35*, disrupted by IS
		*ompk36*, disrupted by IS		*ompk36*, disrupted by IS	*ompk36*, disrupted by IS	*ompk36*, disrupted by IS
Quinolones	GyrA (Ser83Ile, Asp87Gly)	GyrA (Ser83Ile)	GyrA (Ser83Ile)	GyrA (Ser83Ile)	GyrA (Ser83Ile)	GyrA (Ser83Ile)
	ParC (Ser80Ile)	ParC (Ser80Ile)	ParC (Ser80Ile)	ParC (Ser80Ile)	ParC (Ser80Ile)	ParC (Ser80Ile)
	*oqxA*	*oqxA*	*oqxA*	*oqxA*	*oqxA*	*oqxA*
	*oqxB*	*oqxB*	*oqxB*	*oqxB*	*oqxB*	*oqxB*
Polymyxin		*mgrB*, disrupted by IS				
Tetracycline		*tetA*	*tetA*	*tetA*	*tetA*	*tetA*
			*tetD*	*tetD*		

**FIGURE 2:**
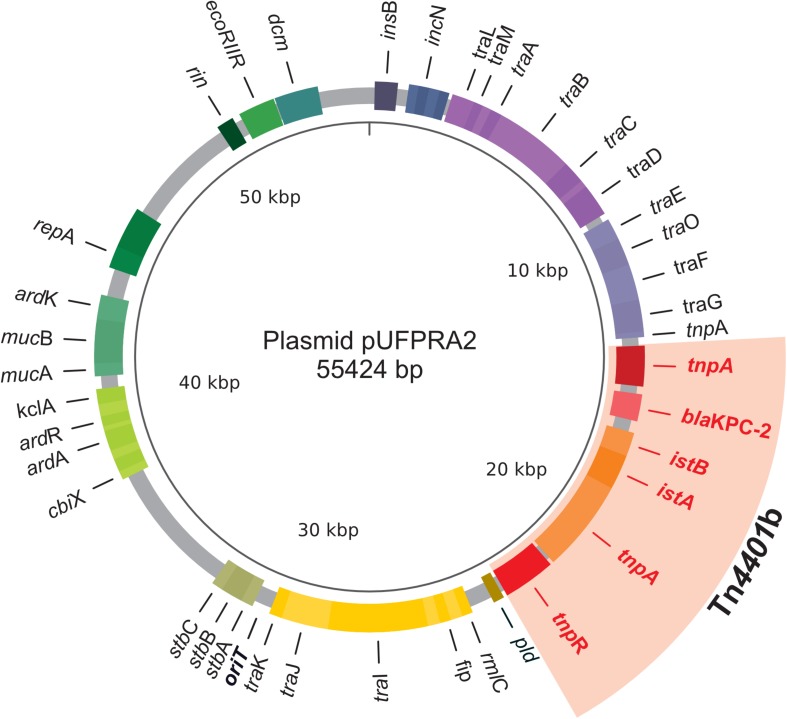
Map of the 55-kb plasmid obtained from KpA2, the index isolate of the KPC outbreak at CHC/UFPR. The representative genes of the pUFPRA2 plasmid are shown in colored boxes. The area in red indicates the Tn*4401b* region. *Tra* genes confirm that it is a conjugative plasmid.

Due to intrinsic methodological limitations, it was not possible to obtain a complete view of the plasmid landscape of each isolate. However, PlasmidSPAdes provided important support for the presence of some plasmids (Supplementary Table 2), including the recovery of a complete conjugative plasmid, pUFPRA2 (Figure 2), which was identified in the index isolates KpA2 and KpA3 of the KPC outbreak, also in KpB10 and KpC2 isolated after the outbreak. This plasmid belongs to the IncN group and carries *bla*_KPC–2_ within a Tn*4401b* transposon. pUFPRA2 possesses 98% identity to pKPC_FCF/3SP (GenBank accession no. CP004367) and 95% identity to pKPC_FCF13/05 (GenBank accession no. CP004366), which are previously published plasmids. The region around ∼15 kbp contains *ardA*, an anti-restriction gene, lacking only in pKPC_FCF13/05. Furthermore, pUFPRA2 presented a Tn*4401b* sequence identical to pKPC_FCF/3SP, including the direct-repeat target site duplications (5′*CTTCAG*3′). We were able to independently recover the complete sequence of plasmid pUFPRA2 from the WGS of all KPC-producing isolates (A2, A3, and C2), although it was not possible to reconstruct the complete plasmid from KpB10 (Supplementary Table 2).

## Discussion

This study describes the gradual increase in antimicrobial resistance in Kp, including an outbreak of KPC and its spread in the hospital between August 2003 and February 2012. Our intention was to study the molecular epidemiology of Kp isolated from a period shift in resistance profile revealed by our hospital infection control service.

Antimicrobial resistance evolution in *Enterobacteriaceae* involved in outbreaks at CHC/UFPR was initially associated with expansion of ESBL-carrying strains co-expressing fluoroquinolone and aminoglycoside resistance genes (Toledo et al., 2012; Nogueira Kda et al., 2014, Nogueira et al., 2015). Since the 2000s, ESBL prevalence has led to an increase in carbapenem prescriptions, resulting in the emergence of ertapenem-resistant strains between 2004 and 2009 (Nogueira Kda et al., 2014, Nogueira et al., 2015).

Several functional studies have investigated the role of porins in antimicrobial resistance (Kaczmarek et al., 2006; Fernandez and Hancock, 2012; Sugawara et al., 2016). Here, we evaluated the distribution of *ompk35*, *ompk36*, and *ompk37* mutations and their correlation with other resistance markers. Our results are also consistent with a previous observation that loss of OmpK35 is more frequent than that of OmpK36, particularly among ESBL producers (Domenech-Sanchez et al., 2003). The higher frequency of OmpK35 loss could be explained by selection for a less permeable outer membrane, as suggested by the recent discovery that OmpK35 allows faster influx of β-lactams than OmpK36 (Sugawara et al., 2016). Considering the reported differences in the impact of each porin on permeability, loss-of-function mutations affecting *ompK35* are expected to be more rapidly fixed than those affecting *ompK36*. Among our samples, we found carbapenem-sensitive strains, although some of them were ESBL producers that lost one of the porins. Kp is extremely versatile, and compensation by other outer membrane proteins or changes in gene expression could explain these different resistance profiles (Garcia-Sureda et al., 2011a; García-Sureda et al., 2011b).

In this study, we observed different mutations in porins among isolates recovered from different body sites of the same patient (P13 and P21). Patient P13 had isolates from CSF that were resistant to a single carbapenem (ertapenem), whereas the isolate from the rectal specimen showed high MICs for ertapenem, imipenem, and meropenem. Similar trends were observed for patient P21 from different sources. Concomitant loss of both OmpK35 and OmpK36 was observed in the isolates that were most resistant to carbapenems. These isolates were also found at body sites that contained abundant and diverse microbiota, which is interesting given the roles of the gut human microbiome in antibiotic resistance (Carlet, 2012). Changes in the gut microbiome, particularly those driven by antibiotics, could silently select for increasingly resistant bacteria. These microorganisms may remain for months in the gut of the carrier or translocate through the gut epithelium, promoting infections and cross-transmission to other patients, resulting in outbreaks that are hard to control.

*Klebsiella pneumoniae* carbapenemase-producing Kp were first described in Brazil in 2006 (Monteiro et al., 2009) and their incidence has significantly increased since that time. In 2010, a great dispersion of *bla*_KPC_ was observed in Brazil (Seki et al., 2011; Pereira et al., 2013), including an outbreak in our hospital (Almeida et al., 2014). During 2011 and 2012, few KPC-producing *Enterobacteriaceae* were found in this same hospital (42 cases in 2 years). However, in 2013, the number of cases doubled, and the co-occurrence of *bla*_KPC_ and *bla*_CTX–M_ was widespread, mainly in the ICU. Interestingly, PFGE analysis showed a major cluster containing isolates recovered between 2003 and 2012, including both non-KPC and KPC-Kp. A previous study, also conducted in our hospital, investigated the distribution of ESBL-producing *Enterobacteriaceae* isolated between 2003 and 2008 (Nogueira et al., 2015). They reported that both Kp and *Enterobacter aerogenes* (recently renamed *Klebsiella aerogenes*) isolates were clustered, but clustering was not observed in *Escherichia coli*. Another study showed that 84% of 129 KPC-Kp isolates from different healthcare facilities in Curitiba belonged to two clusters, isolated between 2010 and 2012 (Arend et al., 2015), suggesting that a predominant lineage of Kp might have spread in the city.

Emerging technologies for rapid identification of resistance determinants, such as WGS, may lead to a shift from traditional AST toward the analysis of genetic elements and discovery of emergent resistance mechanisms. By using this technology, we have found a large and diverse repertoire of resistance genes that accounts for most of the MDR phenotype obtained *in vitro*. The genetic MDR profile has been described by co-existence of beta-lactam (*bla*_KPC_, *bla*_CTX–M_, *bla*_TEM_, *bla*_OXA_), quinolone [*aac*(*6*′)*-Ib-cr*, *qnr*], aminoglycosides (AMEs-coding genes, methylases), tetracyclines (*tet*), sulfonamides (*sul*), and trimethoprim (*dfr*) determinants. These elements are frequently mobilized by a variety of mobile genetic elements (insertion sequences, transposons, and integrons) which are recombined in plasmids and/or chromosomes (Carattoli, 2013; Bi et al., 2015; Mathers et al., 2015; Shankar et al., 2017).

Most of the isolates studied here displayed a single genetic cluster under PFGE analysis and all are members of CG258, predominantly distributed among two different sequence types (ST11 and ST437). Kitchel et al. (2009) showed that all members of a single Kp cluster with more than 80% similarity by PFGE belonged to ST258, corroborating with our findings. Our results also revealed higher-than-expected genotypic diversity of isolates from different body sites of the same patient during a short period of antibiotic therapy, highlighting additional potential challenges for the treatment, diagnosis, and surveillance of MDR bacteria.

The *bla*_KPC–2_-bearing plasmid identified in our Kp isolates (pUFPRA2) was similar to pKPP_FCF13/05 and pKPC_FCF/3SP, which were obtained from two distinct blood cultures of patients infected by Kp. The strain harboring FCF1305-Kp belonged to ST442 and was isolated for the first time in Brazil in 2005 from a patient living in the State of São Paulo; FCF3SP-Kp, also a member of ST442, was isolated in 2009 in the same state (Perez-Chaparro et al., 2014). The KPC-Kp outbreak at our hospital, located further South, in the State of Paraná, occurred in 2010. The presence of very similar plasmids in earlier isolates from the neighboring state of São Paulo indicates that these plasmids are successfully spreading among Kp strains in the Brazilian population.

In summary, our results indicate long-term stability of the same cluster and MLST clonal group of Kp that has been observed in hospitals since the rise of the ESBL endemicity period until the development of resistance to carbapenems, including the *bla*_KPC_ outbreak. A considerable amount of genetic variation, particularly in β-lactams resistance determinants, was observed among isolates. Porin mutations may play an important role in increasing carbapenem MIC. In several cases, they were shown to be even more effective than beta-lactamases at inducing carbapenem resistance. In addition, variation in resistance mechanisms between isolates from the same patient suggests selection and propagation of MDR bacteria in the patient’s body and shows how challenging it is for healthcare teams to control and treat such infections. The remarkable transmissibility coupled with limited therapeutic options to fight MDR isolates drastically reduce the effective control of this pathogen in the nosocomial setting. The integration of WGS technologies and computational analyses with diagnostic procedures can contribute to a better understanding of the co-occurrence of several distinct resistance mechanisms.

## Author Contributions

JP, AG, NV, TV, and LD-C conceived the idea and designed the study. JP carried out the sample collections and performed the wet lab experiments. JP, RdS, MS, H-PA, AG, and NV carried out the genome analysis. JP, RdS, TV, and LD-C interpreted the data and wrote the manuscript. All authors read and approved the final version of the manuscript.

## Conflict of Interest Statement

The authors declare that the research was conducted in the absence of any commercial or financial relationships that could be construed as a potential conflict of interest.

## Footnotes

1 http://em100.edaptivedocs.info

2 http://brcast.org.br/, accessed in January 2018.

3 http://www.eucast.org/resistance_mechanisms

4 https://cge.cbs.dtu.dk/services/KmerFinder

5 https://cge.cbs.dtu.dk/services/MLST

6 https://cge.cbs.dtu.dk/services/PlasmidFinder

7 https://uniprot.org

8 https://cge.cbs.dtu.dk/services/ResFinder
