# Supplementary material for: Molecular Epidemiology of Multidrug-Resistant Klebsiella pneumoniae Isolates in a Brazilian Tertiary Hospital
Source: Front Microbiol. 2019 Jul 23;10:1669. doi: 10.3389/fmicb.2019.01669 (PMC6664048; doi:10.3389/fmicb.2019.01669)
Supplement: Supplementary file 1 [file Table_1.DOC]

***Supplementary Material***

**Molecular epidemiology of multidrug-resistant *Klebsiella pneumoniae* in a Brazilian tertiary hospital**

**Jussara Kasuko Palmeiro^*^, Robson Francisco de Souza, Marcos André Schörner, Hemanoel Passarelli-Araujo, Ana Laura Grazziotin, Newton Medeiros Vidal, Thiago Motta Venancio^*^, Libera Maria Dalla-Costa^*^**

***Correspondence:** Corresponding authors: [jukasuko@gmail.com](mailto:jukasuko@gmail.com), [thiago.venancio@gmail.com](mailto:thiago.venancio@gmail.com), lmdallacosta@gmail.com

**Supplementary table 1.** Primers sequences used for PCR in this study.

| **Genes** | **Sequence (5' - 3')** | **Fragment size (bp)** | **PCR conditions** | **Reference** |
| --- | --- | --- | --- | --- |
| *bla*_CTX-M1_ | F AAAAATCACTGCGCCAGTTC | 415 | 94ºC/ 25 s; 52ºC/ 40 s; 72ºC/ 50 s |  |
|  | R AGCTTATTCATCGCCACGTT |  |  |  |
| *bla*_CTX-M2_ | F CGACGCTACCCCTGCTATT | 552 | 94ºC/ 25 s; 52ºC/ 40 s; 72ºC/ 50 s |  |
|  | R CCAGCGTCAGATTTTTCAGG |  |  |  |
| *bla*_CTX-M8_ | F TCGCGTTAAGCGGATGATGC | 666 | 94ºC/ 25 s; 52ºC/ 40 s; 72ºC/ 50 s |  |
|  | R AACCCACGATGTGGGTAGC |  |  |  |
| *bla*_CTX-M9_ | F CAAAGAGAGTGCAACGGATG | 205 | 94ºC/ 25 s; 52ºC/ 40 s; 72ºC/ 50 s |  |
|  | R ATTGGAAAGCGTTCATCACC |  |  |  |
| *bla*_CTX-M25_ | F GCACGATGACATTCGGG | 327 | 94ºC/ 25 s; 52ºC/ 40 s; 72ºC/ 50 s |  |
|  | R AACCCACGATGTGGGTAGC |  |  |  |
| *bla*_TEM_ | F ATGAGTATTCAACATTTCCG | 840 | 94ºC/ 25 s; 58ºC/ 40 s; 72ºC/ 50 s |  |
|  | R CCAATGCTTAATTCAGTGACG |  |  |  |
| *bla*_SHV_ | F TCAGCGAAAAACACCTTG | 500 | 94ºC/ 25 s; 58ºC/ 40 s; 72ºC/ 50 s |  |
|  | R TCCCGCAGATAAATCACCA |  |  |  |
| *bla*_BES_ | F GTGGTTGCTGGTGGTGATAG | 766 | 94ºC/ 25 s; 58ºC/ 40 s; 72ºC/ 50 s |  |
|  | R CGGGCTGGGTAAAGTAGATG |  |  |  |
| *bla*_GES_ | F ATGCGCTTCATTCACGCA | 860 | 94ºC/ 25 s; 58ºC/ 40 s; 72ºC/ 50 s |  |
|  | R CTATTTGTCCGTGCTCAGG |  |  |  |
| *bla*_PER_ | F GCATCAGGTBGATCAGGGVAA | 550 | 94ºC/ 30 s; 58ºC/ 45 s; 72ºC/ 1 min |  |
|  | R CCGYCCATCAGGCAACAK |  |  |  |
| *bla*_VEB_ | F ACGGTAATTTAACCAGATAGG | 970 | 94ºC/1 min; 46ºC/ 1 min; 72ºC/1 min |  |
|  | R ACCCGCCATTGCCTATGAGCC |  |  |  |
| *bla*_CMY-1-like_ | F GCTGCTCAAGGAGCACAGGAT | 520 | 94ºC/ 30 s; 64ºC/ 30 s; 72ºC/ 1 min |  |
|  | R CACATTGACATAGGTGTGGTGC |  |  |  |
| *bla*_CMY-2-like_ *bla*_LAT_ | F TGGCCAGAACTGACAGGCAAA | 462 | 94ºC/ 30 s; 64ºC/ 30 s; 72ºC/ 1 min |  |
|  | R TTTCTCCTGAACGTGGCTGGC |  |  |  |
| *bla*_DHA_ | F AACTTTCACAGGTGTGCTGGGT | 405 | 94ºC/ 30 s; 64ºC/ 30 s; 72ºC/ 1 min |  |
|  | R CCGTACGCATACTGGCTTTGC |  |  |  |
| *bla*_ACC_ | F AACTTTCACAGGTGTGCTGGGT | 346 | 94ºC/ 30 s; 64ºC/ 30 s; 72ºC/ 1 min |  |
|  | R TTCGCCGCAATCATCCCTAGC |  |  |  |
| *bla*_MIR_ *bla*_ACT_ | F TCGGTAAAGCCGATGTTGCGG | 302 | 94ºC/ 30 s; 64ºC/ 30 s; 72ºC/ 1 min |  |
|  | R TCGGTAAAGCCGATGTTGCGG |  |  |  |
| *bla*_FOX_ | F AACATGGGGTATCAGGGAGATG | 190 | 94ºC/ 30 s; 64ºC/ 30 s; 72ºC/ 1 min |  |
|  | R CAAAGCGCGTAACCGGATTGG |  |  |  |

Continued.

| **Genes** | **Sequence (5' - 3')** | **Fragment size (bp)** | **PCR conditions** | **Reference** |
| --- | --- | --- | --- | --- |
| *bla*_KPC_ | F CTGTCTTGTCTCATGGCC | 680 | 94ºC/ 25 s; 62ºC/ 40 s; 72ºC/ 50 s |  |
|  | R CCTCGCTGTGCTTGTCATCC |  |  |  |
| *bla*_BKC_ | F ACATAATCTCGCAACGGGCG | 941 | 94ºC/ 10 s; 60ºC/ 30 s; 72ºC/ 1 min |  |
|  | R TCGCCGGTCTTGTTCATCAC |  |  |  |
| *bla*_GIM_ | F TCGACACACCTTGGTCTGAA | 477 | 94ºC/ 25 s; 56ºC/ 40 s; 72ºC/ 50 s |  |
|  | R AACTTCCAACTTTGCCATGC |  |  |  |
| *bla*_IMP_ | F GGAATAGAGTGGCTTAATTCTC | 188 | 94ºC/ 25 s; 56ºC/ 40 s; 72ºC/ 50 s |  |
|  | R CCAAACCACTACGTTATCT |  |  |  |
| *bla*_SPM_ | F AAAATCTGGGTACGCAAACG | 271 | 94ºC/ 25 s; 56ºC/ 40 s; 72ºC/ 50 s |  |
|  | R ACATTATCCGCTGGAACAGG |  |  |  |
| *bla*_SIM_ | F TACAAGGGATTCGGCATCG | 570 | 94ºC/ 25 s; 56ºC/ 40 s; 72ºC/ 50 s |  |
|  | R TAATGGCCTGTTCCCATGTG |  |  |  |
| *bla*_VIM_ | F GATGGTGTTTGGTCGCATA | 390 | 94ºC/ 25 s; 56ºC/ 40 s; 72ºC/ 50 s |  |
|  | R CGAATGCGCAGCACCAG |  |  |  |
| *bla*_NDM-1_ | F GGTTTGGCGATCTGGTTTTC | 621 | 94ºC/ 25 s; 56ºC/ 40 s; 72ºC/ 50 s |  |
|  | R CGGAATGGCTCATCACGATC |  |  |  |
| *bla*_OXA-23_ | F GATCGGATTGGAGAACCAGA | 501 | 94ºC/ 25 s; 52ºC/ 40 s; 72ºC/ 50 s |  |
|  | R ATTTCTGACCGCATTTCCAT |  |  |  |
| *bla*_OXA-40/24_ | F GGTTAGTTGGCCCCCTTAAA | 246 | 94ºC/ 25 s; 52ºC/ 40 s; 72ºC/ 50 s |  |
|  | R AGTTGAGCGAAAAGGGGATT |  |  |  |
| *bla*_OXA-51_ | F TAATGCTTTGATCGGCCTTG | 353 | 94ºC/ 25 s; 52ºC/ 40 s; 72ºC/ 50 s |  |
|  | R TGGATTGCACTTCATCTTGG |  |  |  |
| *bla*_OXA-58_ | F AAGTATTGGGGCTTGTGCTG | 599 | 94ºC/ 25 s; 52ºC/ 40 s; 72ºC/ 50 s |  |
|  | R CCCCTCTGCGCTCTACATAC |  |  |  |
| *bla*_OXA-143_ | F TGGCACTTTCAGCAGTTCCT | 149 | 94ºC/ 25 s; 52ºC/ 40 s; 72ºC/ 50 s |  |
|  | R TAATCTTGAGGGGGCCAACC |  |  |  |
| *bla*_OXA-48_ | F TTGGTGGCATCGATTATCGG | 743 | 94ºC/ 25 s; 52ºC/ 40 s; 72ºC/ 50 s |  |
|  | R GAGCACTTCTTTTGTGATGGC |  |  |  |

*Continued.*

| **Genes** | **Sequence (5' - 3')** | **Fragment size (bp)** | **PCR conditions** | **Reference** |
| --- | --- | --- | --- | --- |
| *ompK35* | F TGATGAAGCGCAATATTCTGG | 1030 | 94ºC/ 1 min; 52ºC/ 1 min; 72ºC/ 5 min |  |
|  | R CCAGCCGCTTTGGTGTAAT |  |  |  |
|  | FF AATGAGGGTAATAAATAATGATGAAGC | 1282 | 94ºC/ 1 min; 52ºC/ 1 min; 72ºC/ 5 min |  |
|  | RR CGAGGTTCCATTGTGATTACTG |  |  |  |
| *ompK36* | F CAGCACAATGAATATAGCCGAC | 1148 | 94ºC/ 1 min; 52ºC/ 1 min; 72ºC/ 5 min |  |
|  | R GCTGTTGTCGTCCAGCAGGTTG |  |  |  |
|  | FF TTGTTGGATTATTCTGCATTTTG | 1299 | 94ºC/ 1 min; 52ºC/ 1 min; 72ºC/ 5 min |  |
|  | RR TCTTACCAGGGCGACAAGAG |  |  |  |
| *ompK37* | F CATTCCGCAGAATGAGACGGCAAC | 1381 | 94ºC/ 1 min; 52ºC/ 1 min; 72ºC/ 5 min |  |
|  | R CGACGATGTTATCGGTAGAGATAC |  |  |  |

**REFERENCES**
