# Supplementary material for: Molecular Epidemiology of Multidrug-Resistant Klebsiella pneumoniae Isolates in a Brazilian Tertiary Hospital
Source: Front Microbiol. 2019 Jul 23;10:1669. doi: 10.3389/fmicb.2019.01669 (PMC6664048; doi:10.3389/fmicb.2019.01669)
Supplement: Supplementary file 2 [file Table_2.DOC]

***Supplementary Material***

**Molecular epidemiology of multidrug-resistant *Klebsiella pneumoniae* in a Brazilian tertiary hospital**

**Jussara Kasuko Palmeiro^*^, Robson Francisco de Souza, Marcos André Schörner, Hemanoel Passarelli-Araujo, Ana Laura Grazziotin, Newton Medeiros Vidal, Thiago Motta Venancio^*^, Libera Maria Dalla-Costa^*^**

***Correspondence:** Corresponding authors: [jukasuko@gmail.com](mailto:jukasuko@gmail.com), [thiago.venancio@gmail.com](mailto:thiago.venancio@gmail.com), lmdallacosta@gmail.com

**Supplementary table 2.** Plasmids identified using PlasmidSPades and PlasmidFinder database *^a^*.

| **PlasmidSPades ID** | **Size (bp)** | **Reference** | **Size (bp)** | **Bacteria** | **Identity (%)** | **Coverage (%)** | **Plasmid name** | **Inc group** |
| --- | --- | --- | --- | --- | --- | --- | --- | --- |
| **A2** |  |  |  |  |  |  |  |  |
| NODE_1 | 55424 | NC_021660.2 | 54605 | *Klebsiella pneumoniae* | 99 | 100 | pKPC_FCF/3SP | IncN |
| NODE_3 | 7422 | NC_022376.1 | 8275 | *Salmonella enterica* | 99 | 52 | pST12 |  |
| NODE_5 | 4520 | NZ_CP008845.1 | 3514 | *Klebsiella oxytoca* | 96 | 86 | pKOXM1D |  |
| NODE_9 | 820 | NC_010064.1 | 2004 | *Escherichia coli* | 100 | 41 | pLMO226 |  |
| **A3** |  |  |  |  |  |  |  |  |
| NODE_1 | 54340 | NC_021660.2 | 54605 | *Klebsiella pneumoniae* | 99 | 100 | pKPC_FCF/3SP | IncN |
| NODE_3 | 4512 | NZ_CP008845.1 | 3514 | *Klebsiella oxytoca* | 96 | 86 | pKOXM1D |  |
| NODE_2 | 12933 | NZ_CP013143.1 | 14928 | *Alcaligenes faecalis* | 99 | 40 | pZD02 |  |
| **B10** |  |  |  |  |  |  |  |  |
| NODE_2 | 42307 | NC_021660.2 | 54605 | *Klebsiella pneumoniae* | 99 | 78 | pKPC_FCF/3SP | IncN |
| NODE_7 | 12691 | NC_021660.2 | 54605 | *Klebsiella pneumoniae* | 100 | 21 | pKPC_FCF/3SP |  |
| NODE_9 | 7345 | NC_010499.1 | 6847 | *Salmonella enterica* | 99 | 74 | pUO-SbR3 |  |
| NODE_11 | 4512 | NZ_CP008845.1 | 3514 | *Klebsiella oxytoca* | 96 | 85 | pKOXM1D |  |
| NODE_12 | 3828 | NC_021981.1 | 4198 | *Escherichia coli* | 99 | 53 | pEC386IL |  |
| NODE_16 | 1385 | NC_010064.1 | 2004 | *Escherichia coli* | 99 | 69 | pLMO226 |  |
| NODE_1 | 97654 | NZ_CP011990.1 | 162533 | *Klebsiella pneumoniae* | 99 | 59 | pUHKPC33 | IncFII |
| NODE_3 | 41907 | NZ_CP013942.1 | 52622 | *Cronobacter malonaticus* | 97 | 52 | pCMA2 |  |
| **C2** |  |  |  |  |  |  |  |  |
| NODE_1 | 55088 | NC_021660.2 | 54605 | *Klebsiella pneumoniae* | 99 | 89 | pKPC_FCF/3SP | IncN |
| NODE_5 | 4518 | NZ_CP008845.1 | 3514 | *Klebsiella oxytoca* | 96 | 86 | pKOXM1D |  |
| NODE_8 | 2108 | NC_010064.1 | 2004 | *Escherichia coli* | 100 | 99 | pLMO226 |  |
| **C9** |  |  |  |  |  |  |  |  |
| NODE_1 | 86746 | NC_022522.2 | 167779 | *Salmonella enterica* | 99 | 50 | p1643_10 | IncA/C2 |
| **D8** |  |  |  |  |  |  |  |  |
| NODE_1 | 9391 | NC_018953.1 | 9294 | *Klebsiella pneumoniae* | 99 | 100 | pIGMS32 | ColRNAI |
| NODE_3 | 7163 | NC_002636.1 | 5112 | *Dichelobacter nodosus* | 85 | 90 | pDN1 | IncQ1 |
| NODE_5 | 4278 | DQ019420.1 | 4405 | *Enterobacter cloacae* | 96 | 99 | pQC |  |

*^a^* Rows showing matches to pKPC FCF/3SP are highlighted.
