# Supplementary material for: Molecular Epidemiology of Multidrug-Resistant Klebsiella pneumoniae Isolates in a Brazilian Tertiary Hospital
Source: Front Microbiol. 2019 Jul 23;10:1669. doi: 10.3389/fmicb.2019.01669 (PMC6664048; doi:10.3389/fmicb.2019.01669)
Supplement: Supplementary file 3 [file Table_3.DOC]

***Supplementary Material***

**Molecular epidemiology of multidrug-resistant *Klebsiella pneumoniae* in a Brazilian tertiary hospital**

**Jussara Kasuko Palmeiro^*^, Robson Francisco de Souza, Marcos André Schörner, Hemanoel Passarelli-Araujo, Ana Laura Grazziotin, Newton Medeiros Vidal, Thiago Motta Venancio^*^, Libera Maria Dalla-Costa^*^**

***Correspondence:** Corresponding authors: [jukasuko@gmail.com](mailto:jukasuko@gmail.com), [thiago.venancio@gmail.com](mailto:thiago.venancio@gmail.com), lmdallacosta@gmail.com

**Supplementary table 3.** General genome features of the six *K. pneumoniae* clinical isolates after genome assembly and annotation

| Features | Isolate ID | | | | | |
| --- | --- | --- | --- | --- | --- | --- |
|  | **KpA2** | **KpA3** | **KpB10** | **KpC2** | **KpC9** | **KpD8** |
| Estimate genome size (bp) | 5,202,780 | 5,183,474 | 5,186,575 | 5,246,821 | 5,202,780 | 5,341,303 |
| Genome coverage | 305x | 167x | 269x | 207x | 56x | 239x |
| Number of scaffolds | 27 | 13 | 23 | 35 | 27 | 22 |
| Number of paired-end reads used | 10,917,024 | 5,959,728 | 9,605,538 | 7,488,270 | 2,027,174 | 8,782,152 |
| %GC | 56.95 | 56.76 | 56.91 | 56.84 | 57.21 | 56.68 |
| Predicted genes | 5383 | 5620 | 5419 | 5477 | 5434 | 5932 |
| Predicted coding sequences | 5168 | 5407 | 5203 | 5257 | 5213 | 5711 |
| tRNAs | 82 | 77 | 81 | 82 | 86 | 83 |
| rRNAs | 17 | 12 | 15 | 15 | 14 | 12 |
| Pseudogenes | 115 | 123 | 119 | 122 | 120 | 125 |
