# Supplementary figures and images for: Molecular Epidemiology of Multidrug-Resistant Klebsiella pneumoniae Isolates in a Brazilian Tertiary Hospital
Source: Front Microbiol. 2019 Jul 23;10:1669. doi: 10.3389/fmicb.2019.01669 (PMC6664048; doi:10.3389/fmicb.2019.01669)

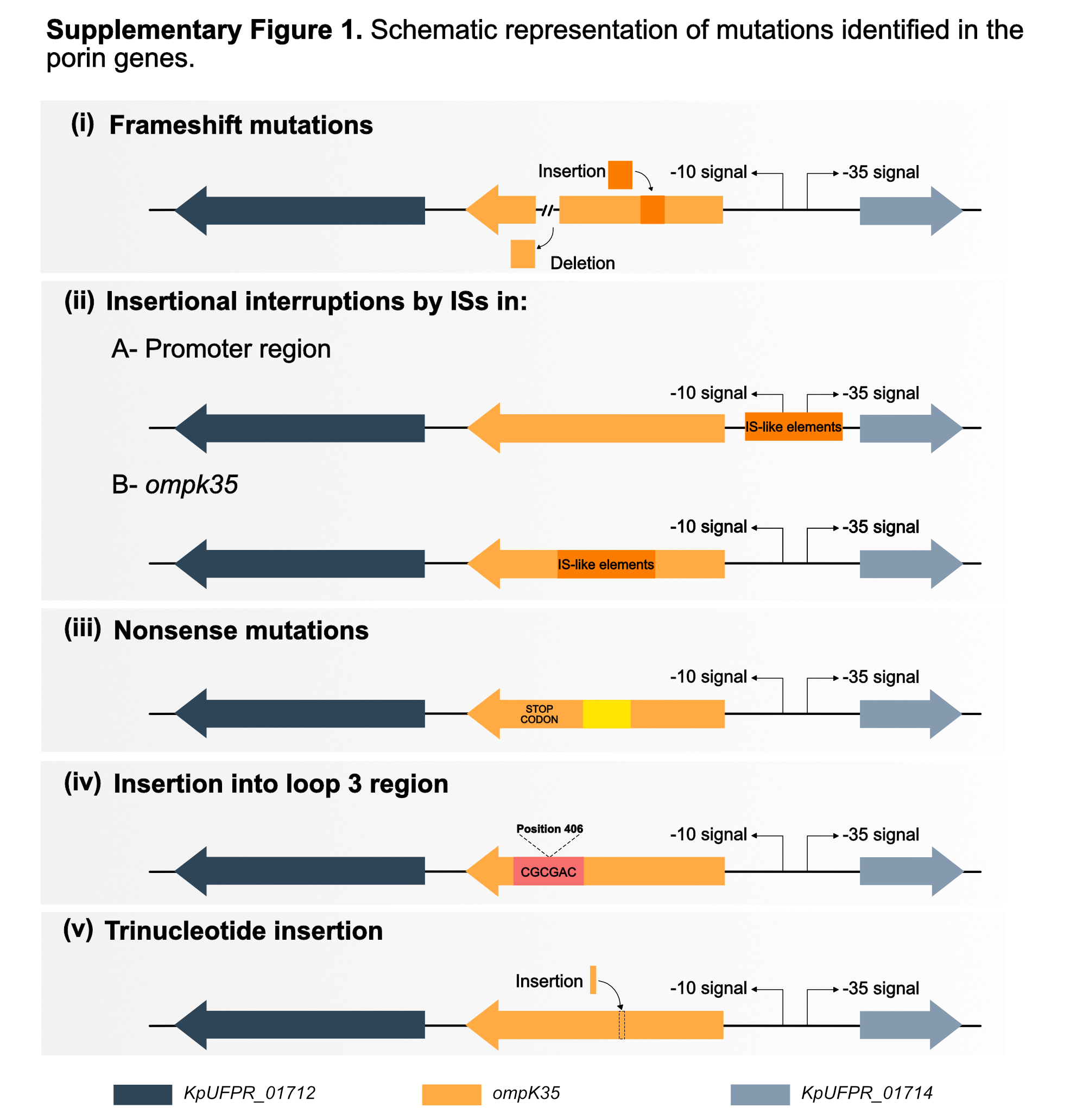

Supplement: Supplementary file 4 [file Image_1.tiff]
